# Comparative Genome Analysis of Two *Streptococcus suis* Serotype 8 Strains Identifies Two New Virulence-Associated Genes

**DOI:** 10.3390/ani14040572

**Published:** 2024-02-08

**Authors:** Qi Sheng, Qiuhua Xu, Zouran Lan, Zongfu Wu

**Affiliations:** 1 MOE Joint International Research Laboratory of Animal Health and Food Safety, College of Veterinary Medicine, Nanjing Agricultural University, Nanjing 210014, China; 2021107071@stu.njau.edu.cn (Q.S.); 2020107073@stu.njau.edu.cn (Q.X.); 2 Key Lab of Animal Bacteriology, Ministry of Agriculture, Nanjing 210014, China; 3 WOAH Reference Lab for Swine Streptococcosis, Nanjing 210014, China; 4 Shandong Provincial Center for Animal Disease Control, Jinan 250100, China; 5 Guangdong Provincial Key Laboratory of Research on the Technology of Pig-Breeding and Pig-Disease Prevention, Guangzhou 511400, China

**Keywords:** *Streptococcus suis* serotype 8, comparative genomics, transposases, virulence factors

## Abstract

**Simple Summary:**

*Streptococcus suis* can cause dangerous infections in swine and humans. Among numerous pathogenic serotypes, serotype 8 is one of the predominant serotypes isolated from different hosts. However, little is known about its pathogenicity and genomic characterization. In this study, we first provided complete genomes of two serotype 8 strains: virulent and non-virulent. Several virulence-associated genes are located in mobile genetic elements that can form circular DNA intermediates, indicating the possibility of horizontal transmission among *S. suis* strains. Mouse infection experiments confirmed the roles of two new virulence-associated genes in *S. suis* virulence. These findings contribute to understanding the genomic characterization of *S. suis* serotype 8 and the pathogenic properties of *S. suis*.

**Abstract:**

*Streptococcus suis* is an important zoonotic pathogen that can cause meningitis and septicemia in swine and humans. Among numerous pathogenic serotypes, *S. suis* serotype 8 has distinctive characteristics such as a high detection rate and causing multi-host infection. There is no complete genome of serotype 8 strains so far. In this study, the complete genome of two *S. suis* serotype 8 strains, virulent strain 2018WUSS151 and non-virulent strain WUSS030, were sequenced. Comparative genomic analysis showed that the homology of the two genomes reaches 99.68%, and the main difference is the distinctive prophages. There are 83 genes unique to virulent strain 2018WUSS151, including three putative virulence-associated genes (PVGs). Two PVGs, *padR* and *marR,* are passenger genes in ISSsu2 family transposons that are able to form circular DNA intermediates during transposition, indicating the possibility of horizontal transmission among *S. suis* strains. The deletion mutant of PVGs *marR* or *atpase* attenuated the virulence of serotype 2 virulent SC070731 in a mouse infection model, confirming their role in *S. suis* virulence. These findings contribute to clarifying the genomic characterization of *S. suis* serotype 8 and *S. suis* pathogenesis.

## 1. Introduction

*Streptococcus suis* is an important pathogen as it can cause severe infections such as meningitis and sepsis in pigs [1], causing severe financial losses to pig farming. *S. suis* is also an emerging zoonotic agent, and it can cause meningitis, sepsis, and toxic shock in humans [2]. It caused two large-scale outbreaks of *S. suis* infection in humans in China in 1998 and 2005, respectively [3]. *S. suis* can be divided into 29 serotypes [4] according to the capsular polysaccharide (CPS) antigens difference. In addition, a novel variant serotype Chz was identified from piglets with acute meningitis in 2015 [5]. Xu et al. obtained the complete sequences of the *cps* loci from non-typeable isolates and identified eight novel *cps* loci (NCLs), designated NCL1 to NCL8, in 2015 [6]. Strains carrying NCL9 to NCL16 were identified in 2016 among 486 isolates collected from pigs in China [7]. Gottschalk et al. identified four new NCLs (NCL17 to NCL20) from 79 non-serotypeable *S. suis* strains in Canada in 2017 [8]. Strains carrying NCL21 to NCL26 were found in 35 non-typeable strains [9]. In 2020, a novel NCL was identified [10]. Thus, a total of 27 NCLs have been identified so far. Among all these strains, *S. suis* serotype 2 is the main one that infects swine and humans [11]. The majority of research so far has been focused on this serotype. However, the characteristics of pathogenicity, population structure, and vehicles for antimicrobial resistance genes vary by serotypes. Serotype 7 strains were clustered into five minimum core-genome (MCG) groups, and integrative and mobilizable elements (IMEs) were the primary vehicle for antibiotic resistance genes transmission [12]. De Greeff et al. found that clinical serotype 9 swine isolates in the Netherlands are genetically similar, whereas serotype 9 strains isolated from healthy pigs have higher heterogeneity [13]. Serotype 9 strains were clustered into seven MCG groups, most of which belonged to MCG groups 1, 3, 4, and 7 [14]. Most serotype 31 strains were clustered to MCG 7-2 and MCG 7-3, and prophages acted as primary vehicles for antimicrobial resistance gene transmission [15], which is different from the previous findings that integrative conjugative elements (ICEs) and IMEs act as major vehicles of antimicrobial resistance genes in *S. suis* [13,16].

*S. suis* serotype 8 is one of the top five predominant serotypes isolated from clinical cases worldwide between 2002 and 2013 [17]. In Spain, the United States, and Canada, serotype 8 strains were ranked fourth among those isolated from diseased pigs [18,19,20]. In the case of *S. suis* clinical isolates from healthy and diseased cattle in Japan, *S. suis* serotype 8 was the most frequently isolated strain [21]. Moreover, serotype 8 has the same capsular structure as *Streptococcus pneumoniae* serotype 19F [17]. The continuous high detection rate and widespread prevalence indicate that *S. suis* serotype 8 is worthy of focus. In our previous study, the population of serotype 8 strains was found to be connected with the geographical distribution and merged into four MCG groups; 15.6% (10/64) of strains belonged to MCG group 1, which was considered to have the capacity to cause global outbreaks; 9 of 12 of representative serotype 8 strains were virulent, and the pathogenic potential of serotype 8 ST1241 strains needed attention [22]. However, no virulence indicators were identified based on reported putative virulence-associated genes [22]. Thus, the new virulence-associated genes need to be investigated.

In this study, we first presented the complete genome of two serotype 8 strains including a virulent strain 2018WUSS151 belonging to ST1241, and a non-virulent strain WUSS030, whose virulence was demonstrated in zebrafish and mice infection models in our previous study [22]. Using a comparative genomic approach, we described the main features of these two genomes and identified two new virulence-associated genes. These findings contribute to understanding the genomic characterization of *S. suis* serotype 8 and the pathogenic properties of *S. suis*.

## 2. Materials and Methods

### 2.1. Bacterial Strains and Cultural Conditions

All bacteria strains used in this study are listed in Table 1. Strain 2018WUSS151 was isolated from a diseased pig in 2018, and strain WUSS030 was isolated from a healthy pig in 2017 [22]. Serotype 2 ST7 virulent strain SC070731 was isolated from a diseased pig [23]. Strains were cultured in Todd-Hewitt broth (THB, Hope Bio-Technology Co., Ltd., Qingdao, China) at 37 °C, or plated on THB agar containing 6% sheep blood at 37 °C and 5% CO_2_. If required, spectinomycin was added to the media at 100 μg/mL concentration. For bacterial growth curve measurements, the overnight cultures were transferred to fresh THB medium at a ratio of 1:100. The total time of the measurement was 12 h with an interval of 2 h.

### 2.2. DNA Extraction

The bacterial genomes were extracted using a Bacterial DNA Kit (TIANGEN, Beijing, China) and quantified using a DS-11^+^ Spectrophotometer (DeNovix Inc., Wilmington, NC, USA).

### 2.3. Sequencing, Assembly, and Annotation of the Genome Sequencing

The complete genomes of strains 2018WUSS151 and WUSS030 were sequenced using Illumina NovaSeq PE150 and PacBio Sequel platform at the Beijing Novogene Bioinformatics Technology Co., Ltd. (Beijing, China). The low-quality reads were filtered (less than 500 bp) and processed to obtain clean data—848,289,626 bp (2018WUSS151) and 838,568,213 bp (WUSS030). The valid sequences were assembled by SMRT Link v5.0.1 [24,25]. CISA [26] was used for data integration. The Arrow tool in SMRT link v5.0.1 [24,25] was used to optimize the preliminary assembly and screen the chromosomes and plasmid sequences to obtain the completion sequence. GeneMarkS (Version 4.17) [27] was used to predict the coding genes of the sequenced genome. The protein sequences of the predicted genes were compared with the NR functional database [28] via Diamond alignment, and the alignment results with the highest score were selected for annotation (default identity ≥ 40%, coverage ≥ 40%). tRNAscan-SE (Version 1.3.1) [29] was used to predict tRNAs; the rRNA was predicted by rRNAmmer (Version 1.2) [30] with the rRNA library alignment of the closely related reference sequences. The complete genomes of strains 2018WUSS151 and WUSS030 were deposited in NCBI (Accessions Nos. NZ_CP101844.1 and NZ_CP110141.1).

### 2.4. Comparative Genomics

The complete genome sequences of strains 2018WUSS151 and WUSS030 were compared and analyzed. OrthoANI [31] was used to explore the affinity of the two strains. Mauve [32] and Circos [33] were used to analyze the genome collinearity and visualize the results.

### 2.5. Analysis of Mobile Genetic Elements (MGEs) and Confirmation of Transposition Mechanism

IslandPath-DIOMB [34] was used to predict Genomics Islands (GIs). Prophages were predicted by PHASTER [35]. Insertion sequences (ISs) were predicted by ISFinder [36]. Several pairs of primers were designed with corresponding passenger genes as target genes, and the PCR technique was used to verify the transposition mechanism of transposons. The primers are listed in Table 2. The terminal inverted repeat of ISs was analyzed and displayed by MEME [37].

### 2.6. Construction of Deletion Mutants

A two-step natural transformation method was applied to construct the deletion mutants [38]. The upstream and downstream knockout genes were amplified to construct the markerless deletion mutants of *padR* (Δ*padR*), *marR* (Δ*marR*), and *atpase* (Δ*atpase*). Overlapping PCR was performed to fuse the upstream, *sacB-spc* cassette, and downstream. Bacteria were grown in THB at 37 °C and 5% CO_2_ until the OD_600_ reached 0.6. Then, the culture was transferred to a fresh THB medium at 1:50 until its OD_600_ reached 0.04–0.08. In total, 100 μL of culture was removed to a new 1.5 mL EP tube, and 5 μL of peptide pheromone was added along with the DNA template mentioned above. The culture was plated on the THB agar plate with spectinomycin (100 μg/mL) after 2 h of incubation. Therefore, the target gene was replaced by the *sacB-spc* cassette after the transformation and selection of spectinomycin. After the second transformation and sucrose selection, the *sacB-spc* cassette was later replaced by a fused upstream and downstream fragment to obtain the markerless deletion mutants. Primers used for the construction of mutant strains are listed in Table 2.

### 2.7. Mice Infections

The SPF BALB/c mice (female, 5-week-old) were purchased from Shanghai Slac Laboratory Animal Co., Ltd. (Shanghai, China). Bacteria were collected at the mid-log phase (OD_600_ = 0.6) and washed twice in PBS. Each mouse (5 mice per group) was intraperitoneally injected with the dose of 3 × 10^8^ CFU (bacterial pellet resuspended in 200 μL PBS) of strain SC070731 (WT), Δ*padR*, Δ*marR*, or Δ*atpase*. Bacterial CFUs of the inoculum were confirmed by plating serial dilutions on THA. At 12 h after infection, all the mice were euthanized. Organ samples, including brain, liver, spleen, and kidney, were collected aseptically and homogenized in PBS. The homogenates with appropriate dilutions were plated on THA to determine the bacterial colonies. To better observe the disease symptoms, the mice infection experiment was reperformed. The infection dose and the number of mice per group are the same as described above. In addition to the above four infection groups (WT, Δ*padR*, Δ*marR*, and Δ*atpase*), group PBS (each mouse injected with 200 μL PBS) was set as a control group.

## 3. Results

### 3.1. Features of Strains 2018WUSS151 and WUSS030 Genomes

**Figure 1:**
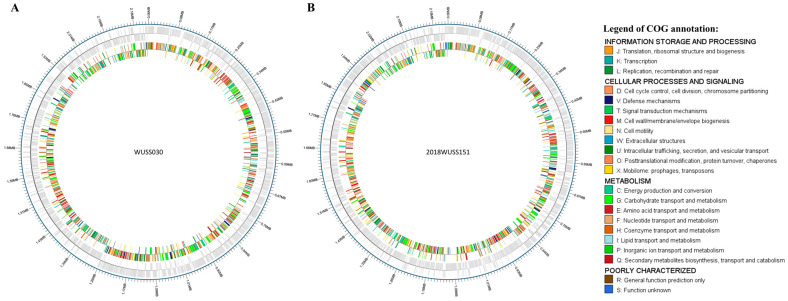
Schematic circular diagrams of strains WUSS030 (**A**) and 2018WUSS151 (**B**) genomes. From outside to inside, the rings represent scale in Mbp, predicted coding genes from two DNA strands (gray), and gene function annotation based on the COG (Clusters of Orthologous Groups) database (color).

The chromosomes of strains WUSS030 (Figure 1A) and 2018WUSS151 (Figure 1B) are 2,223,280 bp and 2,229,493 bp in size, respectively. The genomes of strains WUSS030 and 2018WUSS151 contains 2015 and 2039 predicted protein-coding sequences (CDS), respectively. The chromosomes of the two strains contain different kinds of MGEs, including ICEs, GIs, ISs, transposons, and prophages. It was found that strains 2018WUSS151 and WUSS030 both contain IME_Ssu858_ and ICE_Ssu2018WUSS041_, and IME_Ssu858_ carrying antimicrobial resistance genes *tetO* and *ermB* was inserted into ICE_Ssu2018WUSS041_ [17]. There are no plasmids in these two strains. The summary of the general features of the two genomes is presented in Table 3. The distribution of ISs in genomes of 2018WUSS151 and WUSS030 is displayed in Table 4.

### 3.2. Comparative Genomic Analysis of Strains 2018WUSS151 and WUSS030

**Figure 2:**
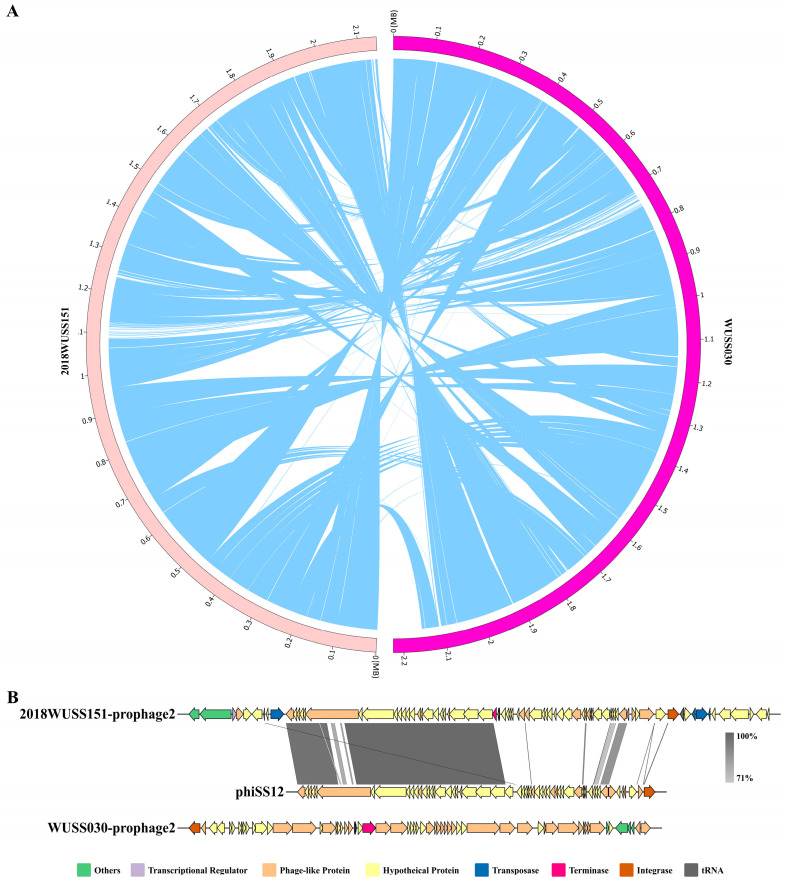
Comparative genome-wide analysis of *S. suis* strains WUSS030 and 2018WUSS151. (**A**) The whole genome sequences of the two strains were compared. The blue ribbons represent the size, position, and orientation of two genomic elements. (**B**) Comparative genome alignments between 2018WUSS151-prophage2 and phiSS12 and annotations of genes in 2018WUSS151-prophage2 and WUSS030-prophage2.

The homology of the genomes of strains 2018WUSS151 and WUSS030 reaches 99.68% (Figure 2A). The main difference between the two genomes is the unique prophages; 2018WUSS151-prophage2 and WUSS030-prophage2 are the two intact prophages within all 12 predicted prophages, and they are distinct from each other. The two prophages contain a large number of hypothetical proteins and phage-like proteins (Figure 2B). The sequence of 2018WUSS151-prophage2 shows nucleotide homology (98% identity, 35% coverage) to *S. suis* prophage phiSS12 obtained from *S. suis* serotype 1/2, indicating horizontal genetic exchange between prophages from different *S. suis* strains. Genes in phiSS12 are grouped into five modules based on the phage life cycle [39], and the 2018WUSS151-prophage2 sequence shares high nucleotide identity with phiSS12, which encodes proteins responsible for lysogeny, packaging, and replication. A total of 83 genes unique to the virulent strain 2018WUSS151 were screened by comparative genomic analysis. Among the 83 specific genes in strain 2018WUSS151, 78 genes are located in MGEs: 52 genes are located in 2018WUSS151-prophage2; 12 genes are located in another predicted prophage; 14 genes are located in the regions between two transposases. Most specific genes encode hypothetical proteins (34/83) and proteins whose functions are related to replication, recombination and repair, defense mechanisms, and transcription. The description of their locations and functions is shown in Supplementary Table S1. Of the 83 specific genes, 3 are putative virulence-associated genes (Table 5).

### 3.3. Transposons Carry Genes Unique to Strain 2018WUSS151

**Figure 3:**
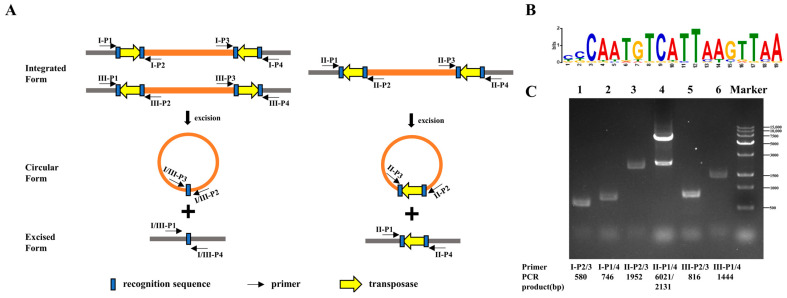
Detection of the forms of transposon circularization. (**A**) The transposase arrangements and circularization forms. Primers are shown as black arrows. Terminal inverted repeats are shown as blue rectangles, and transposases are shown as yellow arrows. The passenger genes are shown as orange rectangles, while others are shown as grey rectangles. (**B**) Terminal inverted repeats. (**C**) The template of all lanes was the genome of 2018WUSS151. The gene locus of passenger genes Ⅰ, Ⅱ and Ⅲ was *NOV99_06305-NOV99_06350*; *NOV99_03505* and *NOV99_08285*, respectively.

**Figure 4:**
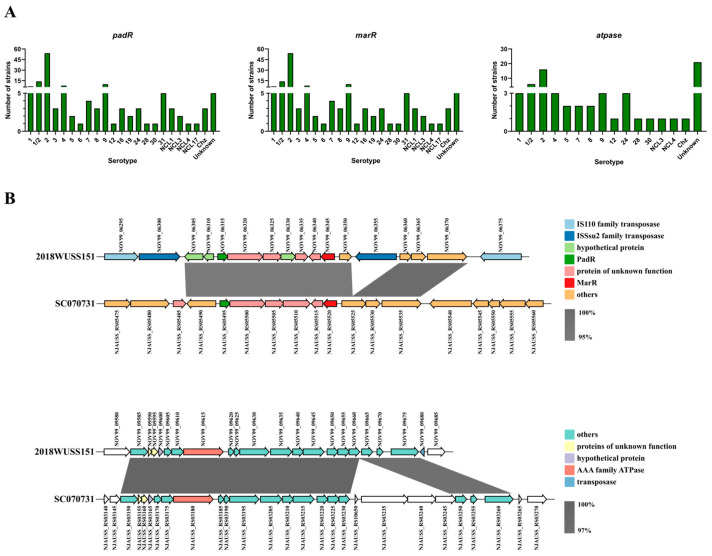
The new virulence-associated genes discovered in strain 2018WUSS151. (**A**) The distribution of three putative virulence-associated genes among complete genomes of *S. suis* strains deposited in NCBI. (**B**) The alignments of putative virulence-associated genes in strains 2018WUSS151 and SC070731.

There are abundant and diverse ISs in strains 2018WUSS151 and WUSS030 genomes. Among them, the IS4-like ISSsu2 family is worth mentioning. The members of the IS4-like ISSsu2 family serve as flanking ISs of transposons, and transposases at both ends are arranged in the same, opposite, or reverse direction (Figure 3A). The terminal inverted repeat sequence of ISSsu2 is CAATGTCATTAAGTTAA (Figure 3B). Two putative virulence-associated genes, *padR* and *marR*, are passenger genes in transposons. PCR analysis confirmed that the transposases arranged in three ways are able to form circular DNA intermediates during transposition (Figure 3C), suggesting that they could be horizontally transmitted among *S. suis* strains. As shown in Figure 4A, three putative virulence-associated genes are widely distributed among 148 complete genomes of *S. suis* strains deposited in NCBI, *padR* (137/148), *marR* (138/148), and *atpase* (67/148). They are also present in 64 serotype 8 strains analyzed in our previous study [22], *padR* (61/64), *marR* (61/64), and *atpase* (29/64), with draft genomes.

### 3.4. The New Virulence-Associated Genes Discovered in Strain 2018WUSS151

To investigate the functions of the putative virulence-associated genes, we tried to construct mutant strains of corresponding genes in strain 2018WUSS151. A series of failed attempts to construct mutant strains in 2018WUSS151 have been made, including using *S. suis*-*E. coli* shuttle plasmid pSET4s [40] and the two-step natural transformation [38]. Since the putative virulence-associated genes were widely distributed in *S. suis* and also present in serotype 2 strain SC070731, which belongs to the highly virulent ST7 type [23], strain SC070731 was chosen to explore the roles of these genes in *S. suis* virulence. As shown in Figure 4B, the putative virulence-associated genes (*padR*, *marR*, and *atpase*) and their surrounding genes in strains 2018WUSS151 and SC070731 show high nucleotide identity (>95%). However, there are no ISs around the putative virulence-associated genes in strain SC070731.

**Figure 5:**
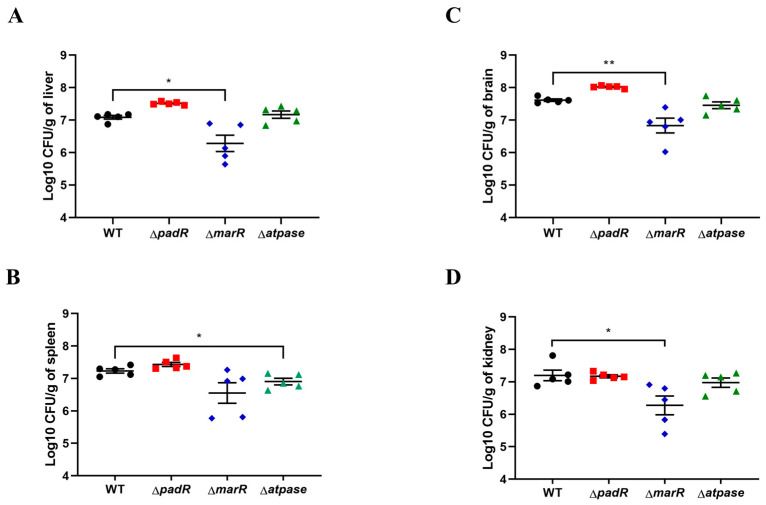
*marR* and *atpase* contribute to *S. suis* virulence in a mouse infection model. At 12 h after infection, bacterial numbers in liver (**A**), spleen (**B**), brain (**C**), and kidney (**D**) tissues were determined. Statistical analysis was performed using a two-tailed unpaired *t* test. “*” indicates *p* < 0.05, and “**” indicates *p* < 0.01.

Mouse infection experiments using WT, ∆*padR*, ∆*marR*, or ∆*atpase* were performed to assess the contribution of the putative virulence-associated genes to *S. suis* virulence. Bacterial growth curve measurements showed no difference in growth among them in the THB medium (Supplementary Figure S1). As shown in Supplementary Figure S2, after 12 h of infection, mice in the PBS control group showed no symptoms. However, those infected with WT and three deletion mutants all appeared to have disease symptoms, including depression and a rough hair coat. Mice were euthanized at 12 h post-infection to analyze viable bacteria from multiple tissues. The bacterial number of ∆*marR* in liver, brain, and kidney tissues was significantly less than that of the WT infection group (Figure 5). The bacterial number of ∆*atpase* in the spleen was significantly lower than that of WT infection groups (Figure 5). The results indicate that ∆*marR* and ∆*atpase* substantially decreased *S. suis* virulence in a mouse infection model, suggesting that *marR* and *atpase* play a role in *S. suis* pathogenicity.

## 4. Discussion

*Streptococcus suis* serotype 8 is one of the most important pathogenic serotypes, and it has a high isolation rate from clinical cases in South America, Asia, and Europe [11]. Serotype switching can occur between *S. suis* strains; Okura et al. reported that the strain changing CPS from serotype 2 to serotype 8 became hyper-virulent [41]. The information above suggests that *S. suis* serotype 8 is non-negligible.

Mobile genetic elements (MGEs) are central to bacterial adaptation and evolution [42,43]. They serve as vehicles for antibiotic resistance genes and key factors for virulence. The strengthening of global swine trade can give rise to genetic variation through the interaction of MGEs, thus contributing to the selective advantage and adaptation of *S. suis* [13]. Li et al. performed the genomic analysis of 1634 *S. suis* isolates and found a novel clade of human-associated *S. suis* (HAC). HAC can be divided into three sub-lineages (I, II, and III), and strains of the three sub-lineages possess an 89 kb, 78 kb, and 127 kb pathogenicity island (PAI), respectively [44]. Therefore, analyzing and understanding the factors like MGEs that may become involved in zoonotic infection and adaptation are crucial to controlling and preventing *S. suis* infection. The analysis of the complete genomes of strains 2018WUSS151 and WUSS030 found various kinds of MGEs, such as ICEs, ISs, prophages, and transposons. Strains 2018WUSS151 and WUSS030 contain ICE_Ssu2018WUSS041_ inserted with IME_Ssu858_, which carries antimicrobial resistance genes *tetO* and *ermB* [22]. This discovery is consistent with the prevalence of antibiotic resistance to macrolides, lincosamides, and tetracyclines in *S. suis* serotype 8 strains [22]. Transposons play a key role in the evolution of host genomes [45] and horizontal gene transfer (HGT) [46]. For instance, transposons carrying antimicrobial resistance genes can facilitate their spread [47]. Transposons also contribute to acquiring new traits [48] and enhance genome stability [49]. Two of the three putative virulence-associated genes mentioned above are passenger genes in transposons, and transposon circular intermediates can be produced during transposition, which indicates that the genes can be horizontally transferred between different hosts. An IS or transposon can be embedded in a conjugative plasmid, an ICE, or a genomic island to realize horizontal gene transfer [46,50]. The classification of IS families is based on the transposases they have. Transposases can be divided into two major types according to the chemistry used during transposition: DDE (or DEDD) and HUH transposases. The IS4 family was one of the first identified IS families in 1977, and it encodes DDE transposases [51]. With the accumulation of additional related ISs, the internal divergence of the IS4 family has been highly elevated [52], while no study has researched the IS4-like ISSsu2 family so far. In addition to the IS4 family, other IS families, including IS3, ISL3, and IS110, are also present in the genomes of the two strains (Table 4). The IS3 family, which encodes DDE transposases, is one of the largest IS families and is widely distributed in more than 270 bacterial species. During transposition, IS3 family members also adopt a mechanism of producing a circular intermediate [45]. The ISL3 family has more than 120 members from almost 80 bacterial species. It was reported that the transposition of ISL3 family members may prefer AT-rich regions, but their transposition mechanism remains unclear [45]. The IS100 family is the only one to encode DEDD enzymes and can be found in nearly 130 bacterial species. IS110 family members do not have terminal inverted repeats and usually will not generate flanking target directly repeated duplication during insertion, which differs from the DDE IS [45]. The IS4 and IS100 family members are far greater in number than members of other IS families in strains 2018WUSS151 and WUSS030, suggesting that those IS families may have been inserted earlier and experienced events like deletion, transposition, and horizontal transfer with the lapse of time. Prophages integrated into the bacterial genomes function as a double-edged sword. They can disrupt gene expression and cause a fitness burden on bacterial cells. On the other hand, prophages have been proven to benefit bacteria in the process of bacterium–phage symbiotic interaction [53]. The functions of many genes in 2018WUSS151-prophage2 and WUSS030-prophage2 remain unclear. Whether the unknown-function genes in prophages and transposons in two *S. suis* serotype 8 strains are involved in adapting multi-hosts is worthy of further research.

In the present study, three genes that differ in functions were selected as putative virulence-associated genes because their homologs are reported to be related to bacterial virulence or stress response. *padR* encodes a transcriptional regulator of the PadR family, which plays a role in virulence regulation and antibiotic efflux in *Vibrio cholerae* and *Lactococcus lactis* [54,55]. In *V. cholerae*, AphA shares amino acid homology (31.1% identity, 71% coverage) with PadR, and it promotes the production of toxin-coregulated pilus and cholera toxin by activating the transcription of the *tcpPH* on the pathogenicity island [54]. *L. lactis* LmrR shows homology (34.04% identity, 83% coverage) to PadR. LmrR regulates the expression of a multidrug ABC transporter via multidrug binding and induction [55]. *marR* encodes a transcriptional regulator of the MarR family. In pathogens such as *Salmonella* and *Staphylococcus*, homologs of MarR family transcriptional regulators act as central regulators of virulence gene expression, responding to small molecule ligands or redox conditions [56]. *atpase* encodes AAA-family ATPase, and several members of the AAA family are related to the toxin–antitoxin system of type IV toxin (NCBI Conserved Domain Database). SPD_0932 in *S. pneumoniae* shares the same conserved domain with ATPase [57]; it promotes the use of mucins and contributes to bacterial colonization and virulence [57]. However, the targets of MarR and the function of MarR and ATPase in *S. suis* pathogenicity deserve further investigation.

## 5. Conclusions

This study first sequenced and analyzed the complete genomes of two *S. suis* serotype 8 strains, 2018WUSS151 and WUSS030, displayed the features of the two genomes, and identified two virulence-associated genes.

## Figures and Tables

**Table 1:** The information on strains or isolates used in this study.

Strains	Origin	NCBI Accession
2018WUSS151	Isolated from a diseased pig	NZ_CP101844.1
WUSS030	Isolated from a healthy pig	NZ_CP110141.1
SC070731	Isolated from a diseased pig	NC_020526.1
Δ*padR*	This study	
Δ*marR*	This study	
Δ*atpase*	This study	

**Table 2:** Primers used in this study.

Primer.	Primer Sequence (5′–3′)	Comment
**Verification the transposition mechanism of transposons**		
I-P1	TGGCAAATCTATCTCTGCAT	
I-P2	AACTACCACGCGAACTTATC	
I-P3	ATTCACCGAGTTGAAGATAC	
I-P4	ATCTAAAAGAGAACCTCCGAAC	
II-P1	GCCGATTTATCAGTAGCCCAT	
II-P2	TACTTCTATCTGATCTTC	
II-P3	GAATGCAAAAACTCCCTC	
II-P4	TACTGATTCCGCTAGCAGGAC	
III-P1	TCCGATATAGATTGGCAGGA	
III-P2	CAGCACAAGCAAATATCG	
III-P3	AACTCCTTCTCCATCGAC	
III-P4	CGTGCTATCGAACTCTACGG	
**Construction of deletion strains**		
∆*padR*-A	AAATCGGAGAAACTAGACAG	Upstream of fusion fragment for ∆*padR*
∆*padR*-B	AAGGAGTTTTCAGCATTATCCAAACTCACCTCTTTATCTTTA	
∆*padR*-C	ATATTCATTCTAATTGGTAATCAGATTATGACACGCGCAGATTATTTG	Downstream of fusion fragment for ∆*padR*
∆*padR*-D	ACTGATGTCCGTACTTGGTTT	
*sacB*-F	GGATAATGCTGAAAACTCCTT	*sacB*-*spc* gene cassette
*spc*-R	AATCTGATTACCAATTAGAATGAATAT	
∆*padR*-E	TTTCCTGCTCTTCATCCAC	Detection of deletion of *padR* gene
∆*padR*-F	TTCTTCAATCTTCGCCGTCA	
∆*padR*-G	ATGTACTACCCCGTATCCTC	Detection of deletion of *padR* gene
∆*padR*-H	AAGCTCCCTTCTATAATTCCG	
∆*marR*-A	TAAAGGCCACAGTTGTACC	Upstream of fusion fragment for ∆*marR*
∆*marR*-B	AAGGAGTTTTCAGCATTATCC TATCTACCTCTTTTGATTGAT	
∆*marR*-C	ATATTCATTCTAATTGGTAATCAGATT TTTTTTGAGAGGAGACATTAT	Downstream of fusion fragment for ∆*marR*
∆*marR*-D	TGTTGCCTACTACCAACCTG	
∆*marR*-E	ATTTAATTGGCTCCATGCTT	Detection of deletion of *marR* gene
∆*marR*-F	TGCCAGTCAAAATAATCTGGG	
∆*marR*-G	ATTACTAAAAGATGCACCCCT	Detection of deletion of *marR* gene
∆*marR*-H	AGGTAGATTTTGCAAGCCAA	
∆*atpase*-A	TAAAGGCCACAGTTGTACC	Upstream of fusion fragment for ∆*atpase*
∆*atpase*-B	AAGGAGTTTTCAGCATTATCC TATCTACCTCTTTTGATTGAT	
∆*atpase*-C	ATATTCATTCTAATTGGTAATCAGATT TTTTTTGAGAGGAGACATTAT	Downstream of fusion fragment for ∆*atpase*
∆*atpase*-D	TGTTGCCTACTACCAACCTG	
∆*atpase*-E	ATTTAATTGGCTCCATGCTT	Detection of deletion of *atpase* gene
∆*atpase*-F	TGCCAGTCAAAATAATCTGGG	
∆*atpase*-G	ATTACTAAAAGATGCACCCCT	Detection of deletion of *atpase* gene
∆*atpase*-H	AGGTAGATTTTGCAAGCCAA	

**Table 3:** Characteristics of the complete genomes of strains 2018WUSS151 and WUSS030.

Strains	Size (bp)	G + C (%)	tRNA	rRNA	sRNA	GI	Prophage	IS
2018WUSS151	2,229,493	41.11	57	12	2	8	6	122
WUSS030	2,223,280	41.17	54	12	3	9	6	118

**Table 4:** The distribution of ISs in strains 2018WUSS151 and WUSS030.

	Families	DDE ^1^									DEDD ^2^	HUH ^3^
Strains		IS3	IS30	ISL3	IS4	IS5	IS630	IS66	ISAs1	IS982	IS110	IS200/IS605
2018WUSS151		5	1	13	28	3	4	3	1	8	51	4
WUSS030		4	1	14	35	4	5	3	1	8	38	4

^1,2,3^ D, E, H, and U represent the amino acids at the catalytic activity center of enzymes: Asp, Glu, His, and a hydrophobic amino acid.

**Table 5:** Putative virulence-associated genes of strain 2018WUSS151.

Number	Genes	Gene Locus	Gene Function
1	*padR*	NOV99_06315	PadR family transcriptional regulator
2	*marR*	NOV99_06345	MarR family transcriptional regulator
3	*atpase*	NOV99_09615	AAA family ATPase

## Data Availability

The data supporting this study’s findings are available from the corresponding author upon reasonable request.

## Supplementary Materials

The following supporting information can be downloaded at: https://www.mdpi.com/article/10.3390/ani14040572/s1, Table S1: Genes specific to *S. suis* serotype 8 virulent strain 2018WUSS151, Figure S1: The growth curves of WT, ∆*padR*, ∆*marR*, and ∆*atpase* cultured in THB medium, Figure S2: The symptoms of mice injected with PBS, strains WT, ∆*padR*, ∆*marR*, or ∆*atpase*.
