# Supplementary material for: Comparative Genome Analysis of Two Streptococcus suis Serotype 8 Strains Identifies Two New Virulence-Associated Genes
Source: Animals (Basel). 2024 Feb 8;14(4):572. doi: 10.3390/ani14040572 (PMC10886379; doi:10.3390/ani14040572)
Supplement: Supplementary file 1 [file animals-14-00572-s001.zip › Supplementary Materials-Table.pdf]

Supplemental Table 1. *Genes specific to S. suis serotype 8 virulent strain 2018WUSS151*

Number	Gene_locus	Location	Gene function	COG function classification
1	NOV99_01990		helix-hairpin-helix domain-containing protein	Replication, recombination and repair
2	NOV99_03030		restriction endonuclease subunit S	Defense mechanisms
3	NOV99_05425	prophage	hypothetical protein	
4	NOV99_05460	prophage	gp58-like family protein	
5	NOV99_05465	prophage	hypothetical protein	
6	NOV99_05470	prophage	hypothetical protein	
7	NOV99_05475	prophage	hypothetical protein	
8	NOV99_05480	prophage	DUF6096 family protein	
9	NOV99_05485	prophage	phage tail protein	
10	NOV99_05490	prophage	hypothetical protein	
11	NOV99_05495	prophage	HK97 gp10 family phage protein	
12	NOV99_05500	prophage	phage head-tail connector protein	
13	NOV99_05505	prophage	hypothetical protein	
14	NOV99_05510	prophage	sugar-binding protein	
15	NOV99_05515	prophage	DUF4355 domain-containing protein	
16	NOV99_05520	prophage	hypothetical protein	
17	NOV99_05525	prophage	hypothetical protein	
18	NOV99_05530	prophage	CPCC family cysteine-rich protein	
19	NOV99_05535	prophage	minor capsid protein	
20	NOV99_05540	prophage	phage portal protein	
21	NOV99_05550	prophage	hypothetical protein	
22	NOV99_05565	prophage	ArpU family transcriptional regulator	
23	NOV99_05570	prophage	hypothetical protein	
24	NOV99_05575	prophage	hypothetical protein	
25	NOV99_05580	prophage	hypothetical protein	
26	NOV99_05585	prophage	hypothetical protein	
27	NOV99_05590	prophage	DUF1642 domain-containing protein	
28	NOV99_05595	prophage	restriction endonuclease subunit S	
29	NOV99_05600	prophage	SAM-dependent methyltransferase	Defense mechanisms
30	NOV99_05605	prophage	DUF1372 family protein	
31	NOV99_05610	prophage	DUF3310 domain-containing protein	
32	NOV99_05615	prophage	RusA family crossover junction endodeoxyribonuclease	Replication, recombination and repair
33	NOV99_05620	prophage	hypothetical protein	
34	NOV99_05625	prophage	hypothetical protein	
35	NOV99_05630	prophage	PD-(D/E)XK nuclease-like domain-containing protein	
36	NOV99_05635	prophage	recombinase RecT	Replication, recombination and repair
37	NOV99_05640	prophage	hypothetical protein	

38	NOV99_05645	prophage	hypothetical protein	
39	NOV99_05650	prophage	hypothetical protein	
40	NOV99_05655	prophage	hypothetical protein	
41	NOV99_05660	prophage	hypothetical protein	
42	NOV99_05665	prophage	ATP-binding protein	
43	NOV99_05670	prophage	replication initiator protein A	
44	NOV99_05675	prophage	hypothetical protein	
45	NOV99_05680	prophage	hypothetical protein	
46	NOV99_05685	prophage	hypothetical protein	
47	NOV99_05690	prophage	helix-turn-helix domain-containing protein	
48	NOV99_05695	prophage	phage antirepressor KilAC domain-containing protein	Mobilome: prophages, transposons
49	NOV99_05700	prophage	transcriptional regulator	
50	NOV99_05705	prophage	helix-turn-helix transcriptional regulator	Transcription
51	NOV99_05710	prophage	ImmA/IrrE family metallo-endopeptidase	
52	NOV99_05715	prophage	DUF4041 domain-containing protein	
53	NOV99_05720	prophage	Abi family protein	
54	NOV99_05725	prophage	site-specific integrase	Replication, recombination and repair
55	NOV99_06305	transposon	hypothetical protein	
56	NOV99_06310	transposon	hypothetical protein	
57	NOV99_06315	transposon	PadR family transcriptional regulator	Transcription
58	NOV99_06320	transposon	DUF4097 family beta strand repeat-containing protein	
59	NOV99_06325	transposon	DUF1700 domain-containing protein	Function unknown
60	NOV99_06330	transposon	hypothetical protein	
61	NOV99_06335	transposon	DUF4097 domain-containing protein	
62	NOV99_06340	transposon	DUF1304 domain-containing protein	Function unknown
63	NOV99_06345	transposon	MarR family transcriptional regulator	Transcription
64	NOV99_06350	transposon	threonine/serine exporter family protein	Function unknown
65	NOV99_08285	transposon	SpaH/EbpB family LPXTG-anchored major pilin	
66	NOV99_08290	transposon	SpaA isopeptide-forming pilin-related protein	
67	NOV99_08530	transposon	ribbon-helix-helix domain-containing protein	
68	NOV99_09460		reverse transcriptase domain-containing protein	Mobilome: prophages, transposons
69	NOV99_09615		AAA family ATPase	
70	NOV99_09675		recombinase family protein	Replication, recombination and repair
71	NOV99_09695	transposon	IS110 family transposase	Mobilome: prophages, transposons

72	NOV99_10470	prophage	hypothetical protein	Mobilome: prophages, transposons
73	NOV99_10475	prophage	hypothetical protein	
74	NOV99_10480	prophage	hypothetical protein	
75	NOV99_10485	prophage	hypothetical protein	
76	NOV99_10490	prophage	hypothetical protein	
77	NOV99_10495	prophage	hypothetical protein	
78	NOV99_10500	prophage	HK97 gp10 family phage protein	
79	NOV99_10505	prophage	phage head-tail adapter protein	
80	NOV99_10510	prophage	hypothetical protein	
81	NOV99_10515	prophage	hypothetical protein	
82	NOV99_10520	prophage	P22 coat protein - protein 5 domain protein	
83	NOV99_10525	prophage	phage scaffolding protein	
