# Supplementary material for: Comparative Genome Analysis of Two Streptococcus suis Serotype 8 Strains Identifies Two New Virulence-Associated Genes
Source: Animals (Basel). 2024 Feb 8;14(4):572. doi: 10.3390/ani14040572 (PMC10886379; doi:10.3390/ani14040572)
Supplement: Supplementary file 1 [file animals-14-00572-s001.zip › Supplymentary Materials-Figures 1.18.pdf]

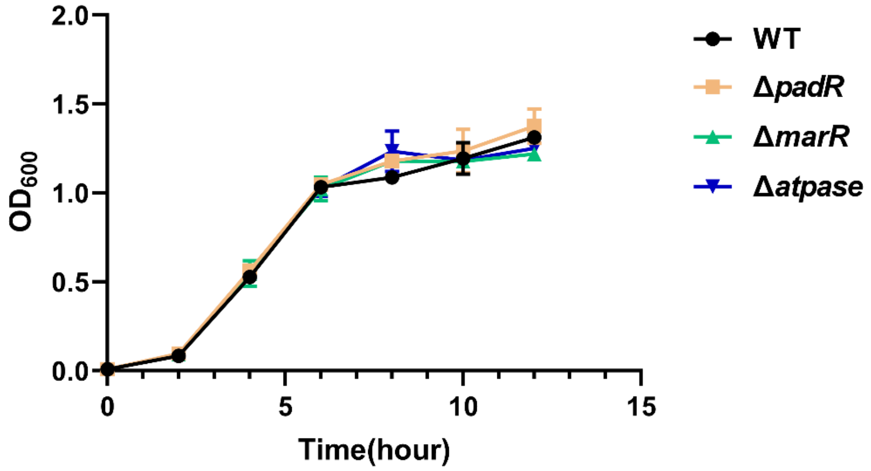

Supplemental Figure 1. The growth curves of wild-type strain (WT) and mutant strains Δ*padR*, Δ*marR*, and Δ*atpase* cultured in THB. The overnight cultures were transferred to fresh THB medium at the ratio of 1: 100. The total time of the measurement was 12 hours with the interval of 2 hours.

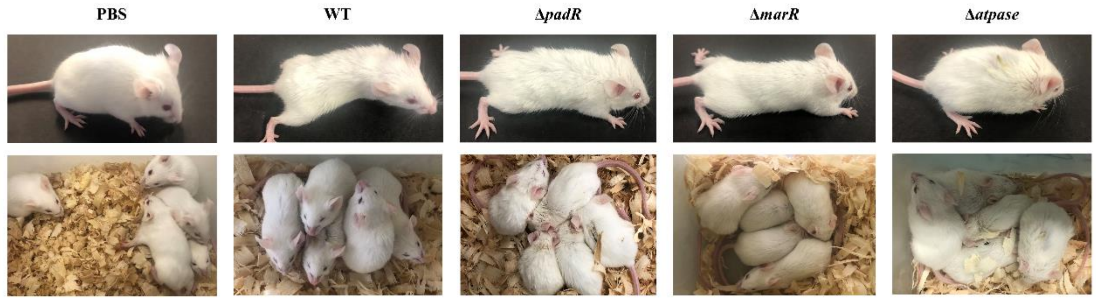

Supplemental Figure 2. The symptoms of mice injected with PBS, WT, Δ*padR*, Δ*marR*, or Δ*atpase*. The symptoms were observed and analyzed at 12 hours after infection. Compared with PBS injection group, mice injected with WT, Δ*padR*, Δ*marR*, or Δ*atpase* showed typical disease symptoms like depression and rough hair coats.
